# A perspective on Alzheimer’s disease: exploring the potential of terminal/paradoxical lucidity and psychedelics

**DOI:** 10.1186/s13024-024-00761-5

**Published:** 2024-10-12

**Authors:** Cong Lin, Xiubo Du, Xiaohui Wang

**Affiliations:** 1 Laboratory of Chemical Biology, Changchun Institute of Applied Chemistry, Chinese Academy of Sciences, Changchun, 130022 China; 2 College of Life Sciences and Oceanography, Shenzhen University, Shenzhen, 518060 China

Alzheimer’s disease (AD) remains a formidable challenge in the field of neurodegenerative disorders, characterized by an insidious onset of memory impairment and a gradual cognitive decline. The molecular pathologies underlying AD are complex and multifactorial, involving a combination of genetic, biochemical, and immunological factors that contribute to its progression [1, 2]. The challenges in treating AD are exacerbated by the molecular complexity of the disease, which has hindered the development of target-based therapeutics. Most existing medications are primarily beneficial only in the early stages of AD, where they can slow the disease’s progression. However, a significant treatment gap exists for late-stage AD, characterized by extensive neuronal damage and severe cognitive decline [3]. This extensive damage complicates efforts to reverse or significantly improve symptoms, posing a major challenge in developing effective interventions for this advanced stage.

Recent observations of terminal/paradoxical lucidity in patients with severe dementia have challenged the longstanding belief that cognitive decline in AD is irreversible. Terminal/paradoxical lucidity refers to unexpected episodes in which individuals with severe dementia temporarily regain cognitive abilities, such as clear communication, emotional expression, and memory recall, typically occurring shortly before death [4]. A recent study indicates that insights into the basis of terminal/paradoxical lucidity may be enhanced by the possibility of regional fluctuations in amyloid-β (Aβ) oligomerization occurring on the appropriate timescale, as shown by cyclic azapeptide oligomer positron emission tomography (PET) ligands. Unlike the continuous amyloid accumulation seen with standard fibrillar amyloid PET, the oligomer tracer shows fluctuations over time without a clear pattern. At certain moments, the ligand illuminates the parietal cortex, but later that area becomes inactive while another region becomes active [5]. Traditionally, it has been thought that once neural pathways are damaged in AD, the decline is permanent due to irreparable pathway damage. However, terminal lucidity suggests that cognitive decline might be reversible, at least momentarily. This phenomenon is unlikely to result from the repair of damaged pathways, as previously assumed in dementia research. Instead, it seems more plausible that these lucidity episodes arise from the spontaneous formation of neural bypasses. These bypasses could temporarily restore connectivity at the network level, facilitating a transient resurgence of cognitive functions in patients with severe dementia [6]. Evidence suggests that it is possible to establish new pathways or circuits, with even silent synapses serving as potential starting points, to circumvent damaged areas and temporarily restore original functions. The abundance of silent synapses in the adult cortex was found to be significantly higher, by an order of magnitude, than previously believed. These synapses can be unsilenced through Hebbian plasticity, recruiting new active connections into a neuron’s input matrix [7]. The development of neural bypasses just before death may be primarily driven by hypoxia. As individuals approach the end of life, often due to organ failure, the oxygen concentration in the central nervous system decreases below normal levels. This reduction in oxygen can cause neurons to become more active, potentially strengthening previously weak connections and forming new neural circuits [8]. These changes in neural activity can lead to hallucinations, out-of-body experiences and altered states of consciousness. These experiences are often interpreted by those experiencing them as spiritual or mystical.

Insights gained from terminal/paradoxical lucidity suggest that even in the late stages of neurodegenerative diseases, the brain retains a latent capacity for functional reorganization. Recently, there has been a resurgence of interest in psychedelics for their potential to rewire the brain, demonstrating significant translational potential in AD. N, N-dimethyltryptamine (DMT), a natural hallucinogen, reduced Aβ accumulation in the hippocampus and prefrontal cortex and improved cognitive impairment by restoring the crosstalk between the endoplasmic reticulum and mitochondria mediated by Sigma-1 receptor [9]. Psychedelics like lysergic acid diethylamide (LSD) and psilocybin have attracted attention for their ability to induce changes in neuroplasticity-the brain’s capacity to form and reorganize synaptic connections. Research indicates that these substances enhance neuroplasticity by promoting the growth of dendritic spines and synapses, and by elevating levels of brain-derived neurotrophic factor (BDNF) [10]. This enhancement is critical for learning and adaptation. Additionally, psychedelics influence meta-plasticity, or the ‘plasticity of plasticity,’ which regulates the thresholds for synaptic strengthening or weakening in response to environmental factors and neural activity. This modulation can effectively ‘reopen’ critical periods of brain plasticity—windows that are typically only open during early development when the brain is highly receptive to environmental stimuli [11]. This aspect is particularly important considering that patients with severe dementia are often of advanced age and exhibit significantly reduced neuroplasticity and learning capabilities. By reopening these windows, psychedelics facilitate a state of heightened plasticity [12], offering a unique opportunity for behavioral interventions and learning training in patients with severe dementia to form new neural pathways/circuits that restore or replicate original functions. This adaptability is crucial for learning and memory and may be harnessed therapeutically in conjunction with behavioral and learning strategies to mitigate some of the cognitive declines seen in AD (Fig. 1). Therefore, the potential of psychedelics to induce lasting changes after just a single dose or a few doses, when combined with structured learning and behavioral training, could represent a paradigm shift in the treatment of mental health conditions. Additionally, depression is common in AD patients, and traditional treatments often offer limited efficacy [13]. Psychedelics may help by creating a temporary state of increased brain connectivity and flexibility, allowing patients to experience emotional breakthroughs, improve mood, and alleviate depressive symptoms. Studies indicate that psychedelics, when combined with psychotherapy, can induce positive emotional states and facilitate psychological healing, which is crucial for patients struggling with depression linked to cognitive decline. However, while early research is promising, further research is needed to fully understand the safety, efficacy, and potential risks of using psychedelics in patients with AD-related depression.

Microdosing psychedelics is being explored as a potential treatment for AD, offering an innovative approach that involves taking small, sub-perceptual doses of these substances [14]. Unlike full doses, microdosing does not produce hallucinations or significant changes in consciousness but may still positively affect brain function. The potential benefits of microdosing for AD include enhanced neuroplasticity, improved brain connectivity, and greater cognitive flexibility. These effects could help mitigate cognitive decline in AD patients by supporting learning, memory, and overall brain health. Additionally, microdosing may relieve depression, anxiety, and mood disturbances often associated with AD, improving emotional well-being without the intense effects of higher psychedelic doses. However, current clinical data on the safety and efficacy of microdosing for AD and AD-related depression is limited. Further research is needed to determine the optimal dosage, long-term effects, and overall safety of this treatment approach.

**Fig. 1:**
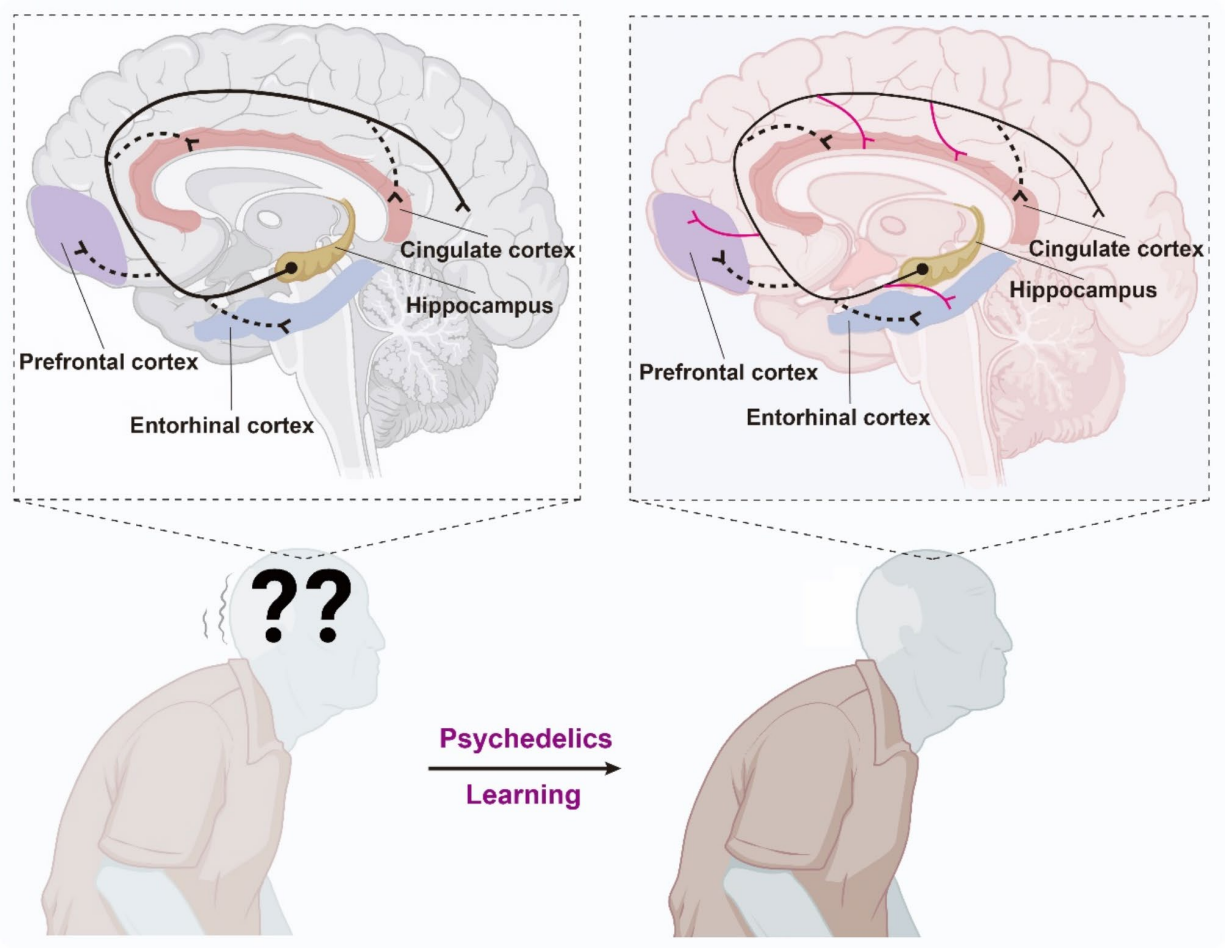
The potential of psychedelics in neural bypass as a therapeutic approach for AD. Psychedelics may temporarily enhance an induced “metaplasticity,” which is believed to establish the critical periods for learning and memory. This effect could combine behavioral and learning strategies to create network-level connectivity, reducing cognitive decline in Alzheimer’s patients. Created by BioRender

Although psychedelics are currently in clinical trials (NCT04123314, NCT01608217 and NCT01302340), integrating them into mainstream clinical practice for dementia presents multiple challenges. Scientifically, the primary hurdle is establishing robust clinical evidence through well-designed trials to verify the efficacy and safety of psychedelics in dementia patients, who may response differently to these substances compared to the general population. Ethically, concerns arise around consent, as patients with dementia might have impaired decision-making capacity; ensuring informed consent and safeguarding patient autonomy are paramount. Additionally, the potential for psychedelic experiences to induce distress or harm must be carefully managed through controlled settings and professional oversight. Regulatory challenges are also significant, as current drug laws classify many psychedelics as controlled substances with high abuse potential and no medical value. Changing these classifications requires substantial evidence from clinical research and concerted efforts to shift public and political perspectives on the therapeutic potential of psychedelics. Altogether, while the promise of psychedelics in enhancing neuroplasticity and cognitive function in dementia is compelling, navigating these hurdles requires a careful, multidisciplinary approach to ensure ethical integrity and regulatory compliance.

In conclusion, the phenomena of terminal/paradoxical lucidity and the emerging research into the use of psychedelics provide groundbreaking insights into the potential reversibility and plasticity of cognitive decline in AD. These observations not only challenge our existing paradigms of neurodegeneration but also open up innovative pathways for treatment that could significantly enhance the quality of life for individuals with AD. By exploring the underlying neural mechanisms and the effects of psychedelics on brain function, we are stepping beyond traditional approaches to unlock novel strategies that could lead to substantive advances in managing AD, particularly in its advanced stages.

## Data Availability

Not applicable.

## Abbreviations

AD: Alzheimer’s disease
LSD: Lysergic acid diethylamide
BDNF: Brain-derived neurotrophic factor
DMT: N, N-dimethyltryptamine
Aβ: Amyloid-β
PET: Positron emission tomography
